# Epidemiological Profile, Clinical Characteristics, and Management of Ovarian Cancer in Eastern India: A Hospital-Based Study

**DOI:** 10.7759/cureus.101937

**Published:** 2026-01-20

**Authors:** Archana Barik, Vinita Singh, Mousumi D Ghosh, Anisha Choudhary, Preeti Yadav

**Affiliations:** 1 Obstetrics and Gynaecology, Manipal Tata Medical College, Manipal, IND; 2 Obstetrics and Gynaecology, Tata Main Hospital, Jamshedpur, IND

**Keywords:** clinical presentation, eastern india, epidemiology, management pattern, ovarian cancer

## Abstract

Introduction: Ovarian cancer (OC) remains a major global health burden. India reports the second-highest number of cases worldwide, with a steadily rising age-standardized incidence rate. Delayed diagnosis, often at advanced stages, is driven by socioeconomic disparities, limited awareness, and nonspecific early symptoms. This study aimed to describe the epidemiological profile, clinical characteristics, and management patterns of OC in Eastern India.

Methods: This retrospective, hospital-based epidemiological study was conducted at Tata Main Hospital, Jamshedpur, India, between January 2019 and December 2024. A sample of 130 patients with histopathologically confirmed primary OC was included. Sociodemographic variables, reproductive and medical history, clinical presentation (International Federation of Gynecology and Obstetrics (FIGO) stage, symptoms), tumor markers (cancer antigen-125 (CA-125), Risk of Malignancy Index (RMI)), histopathology, and treatment modalities were extracted from hospital records. Data were analyzed using descriptive statistics.

Results: The median age at presentation was 50.5 years. Half of the patients belonged to the lower-middle socioeconomic class (65/130, 50.0%). Nulliparity was observed in 13 patients (10.0%), and 62 patients (47.7%) were postmenopausal. A family history of malignancy was present in 11 patients (8.5%). Advanced disease at presentation was common, with FIGO stage III in 51 patients (39.2%) and stage IV in 28 patients (21.5%). Abdominal pain and distension were reported by approximately 94% of patients. Median CA-125 level was 552.5 U/mL, with elevated levels (>35 U/mL) in 95% of patients. Median RMI was 1881, and 93% had RMI >200. Epithelial OC constituted 114 cases (87.7%), with serous adenocarcinoma being the most frequent subtype (76/130, 58.5%). Cytoreductive surgery alone was performed in 25 patients (19.2%), neoadjuvant chemotherapy followed by interval debulking in 48 patients (36.9%), and palliative chemotherapy in 33 patients (25.4%). Fertility-sparing surgery was undertaken in 13 patients (10.0%).

Conclusion: Our findings highlight the high incidence of late-stage OC presentation in Eastern India, potentially linked to socioeconomic factors. The study confirms the utility and effectiveness of established diagnostic markers and adherence to standard management protocols. Future efforts should focus on targeted public health initiatives for early diagnosis, particularly among underserved socioeconomic groups, and multi-institutional studies to validate these regional findings and improve equitable care.

## Introduction

Ovarian cancer (OC) is the eighth most commonly diagnosed malignancy among women globally and remains one of the leading causes of gynecological cancer-related mortality [1]. Substantial geographic variation exists, with higher incidence rates reported in Europe and lower rates in African regions. The global burden of OC is projected to increase significantly by 2040, particularly in countries with a lower Human Development Index (HDI) [2].

In India, OC incidence has been steadily increasing, with population-based cancer registries reporting an annual rise in age-standardized rates (ASR) ranging from 0.7% to 2.4% [3]. India currently reports the second-highest number of OC cases worldwide, following China, with an ASR of 6.6 per 100,000 women [4]. Regional disparities are evident, with higher rates in metropolitan areas and select northeastern states [5]. The age-specific incidence increases sharply after 35 years, peaking between 55 and 64 years [5]. However, these reported figures likely underestimate the true disease burden, as many cases remain underdiagnosed and unreported due to limitations in case detection and reporting systems.

A major challenge in OC management in India is its late presentation, as approximately 70-80% of patients are diagnosed at advanced stages [6]. This is attributed to nonspecific early symptoms, lack of effective screening tools, limited awareness, and socioeconomic barriers to healthcare access. Although transvaginal ultrasonography and serum cancer antigen 125 (CA-125) are commonly used, their low specificity limits their utility for population-based screening [7]. The consequence of late-stage diagnosis is a dismal overall survival rate, with India recording the highest OC mortality globally in 2022 [4].

Among the various histological subtypes of OC, serous epithelial carcinomas consistently exhibit the highest ASRs across most countries, including India, with the mucinous type typically being the second most common [2]. Histological distribution varies with age; epithelial tumors predominate in postmenopausal women, whereas germ cell tumors are more common in younger age groups [8]. Therefore, understanding the age of presentation and histological type is paramount for guiding treatment planning and prognostication.

Multiple risk factors have been associated with OC, including increasing age, nulliparity, infertility, family history, genetic mutations, endometriosis, obesity, and hormonal factors [9]. Hence, a comprehensive understanding of local epidemiology, including prevalent risk factors, is crucial for developing targeted awareness campaigns aimed at reducing exposure to potential risks and facilitating earlier detection.

Current treatment options for OC largely focus on surgical cytoreduction and systemic chemotherapy [10]. The specific sequence and extent of these modalities are dictated by disease stage and the patient's performance status. Complex surgical procedures necessitate specialized skills and experience typically provided by gynecologic oncology surgeons. Evidence supports that appropriate and timely referral to a gynecologic oncologist significantly improves survival outcomes. Enhanced awareness among local practitioners regarding OC epidemiology and high-risk factors is thus essential to ensure prompt referral to specialized cancer centers.

Given the projected rise in OC rates in India, its emergence as a significant public health concern is undeniable. However, a notable gap exists in detailed studies from Eastern India. Therefore, this study aims to delineate the demographic profile, clinical characteristics, histopathological spectrum, and management patterns of OC patients at a tertiary care center in this region.

## Materials and methods

Study design and setting

This was a retrospective, hospital-based epidemiological study conducted at Tata Main Hospital, a tertiary-care, multispecialty hospital located in Jamshedpur, an industrial city belonging to the state of Jharkhand, India. This hospital caters to a large population from surrounding rural and urban areas and is a referral center for many health centers scattered over the state of Jharkhand and nearby states. The study period spanned from January 2019 to December 2024, aiming to investigate the epidemiological factors of OC within the recruited patient population during this defined timeframe.

Study population

The study population comprised patients identified from hospital medical records and pathology databases at Tata Main Hospital during the period from January 2019 to December 2024.

Inclusion and exclusion criteria

All patients with a histopathologically confirmed diagnosis of primary OC, established by biopsy and/or surgical resection specimens, were included. Both newly diagnosed cases during the study period and patients diagnosed prior to the study period who presented to the hospital for treatment or follow-up within the defined timeframe were eligible, provided their medical records contained complete demographic, clinical, and pathological data required for analysis. Patients were excluded if the diagnosis was not confirmed on histopathology, if essential medical records were incomplete or missing, if the ovarian involvement represented metastatic disease from non-ovarian primary malignancies (such as gastrointestinal or breast cancers), or if the tumors were benign or borderline in nature without definitive malignant histopathological confirmation.

Data collection

Data were systematically extracted from existing patient records and hospital databases. This comprehensive collection encompassed sociodemographic characteristics, including age, gender, marital status, educational attainment, occupation, and socioeconomic status. The modified Kuppuswamy scale was used for grading socioeconomic status [11]. Detailed medical history was gathered, covering parity, age at menarche and menopause, documented hormonal therapy use (such as oral contraceptives and hormone replacement therapy), recorded family history of ovarian or related cancers (including breast and colorectal), and any noted gynecological conditions (e.g., endometriosis and polycystic ovarian disease). Furthermore, comprehensive clinical and diagnostic data were meticulously collected, including presenting symptoms and their duration, the International Federation of Gynecology and Obstetrics (FIGO) stage at diagnosis [12], histological subtype, tumor grade, presence of ascites, CA-125 levels, and the calculated Risk of Malignancy Index (RMI) score [13]. Relevant treatment history, past medical conditions, reproductive history (documented pregnancies, breastfeeding periods, and tubal ligation status), and lifestyle elements (smoking, alcohol consumption, dietary habits, and physical activity) were also investigated. All OC diagnoses were definitively confirmed through histopathological examination of biopsy or operative specimens, with all pathological reports meticulously reviewed. Data extracted from hospital databases included all cases of ovarian malignancy admitted during the study period. No missing or incomplete data were identified for the variables included in the analysis, as records were comprehensively maintained and verified.

Data management and statistical analysis

All collected data were entered into Microsoft Excel for Microsoft 365 (Microsoft Corporation, Redmond, WA, USA) and were double-checked for accuracy and completeness by the principal investigator to minimize entry errors. Statistical analysis was performed using EPI Info™ version 7.2 (Centers for Disease Control and Prevention, Atlanta, USA). Descriptive statistics were used to summarize the study population. Continuous variables were expressed as median with IQR, while categorical variables were presented as frequencies and percentages. Associations between categorical variables were assessed using the chi-square test, with a p-value<0.05 considered statistically significant.

Ethical consideration

Ethical approval was obtained from the Institutional Ethics Committee of Tata Main Hospital, and a waiver of informed consent was granted for the use of de-identified data, in accordance with institutional guidelines for retrospective research.

## Results

A total of 130 patients were included. The median (IQR) age was 50.5 (42-61.6) years, with most patients aged between 40 and 59 years. Lower-middle socioeconomic status was observed in 65 patients (50.0%) (Table 1).

**Table 1:** Sociodemographic characteristics of the study participants (n=130)

Variable	Category	No. of patients	Percentage (%)
Age (years)	<19	5	3.8
	20-29	7	5.4
	30-39	17	13.1
	40-49	33	25.4
	50-59	33	25.4
	60-69	20	15.4
	70-79	12	9.2
	≥80	3	2.3
Body mass index (kg/m²)	≤18.5 (underweight)	5	3.8
	18.5-24.9 (normal weight)	114	87.7
	25-29.9 (overweight)	11	8.5
	≥30 (obese)	0	0.0
Socio-economic class (Modified Kuppuswamy scale)	Upper (I)	4	3.1
	Upper-middle (II)	30	23.1
	Lower-middle (III)	65	50.0
	Upper-lower (IV)	14	10.8
	Lower (V)	17	13.1

Nulliparity was present in 13 patients (10.0%), and 62 patients (47.7%) were postmenopausal. Breastfeeding history was reported by 117 patients (90.0%), while oral contraceptive pill (OCP) use was reported by 10 patients (7.7%) (Table 2).

**Table 2:** Obstetric and gynecologic parameters of the study participants (n=130)

Variable	Category	No. of patients	Percentage (%)
Parity	Nulliparous	13	10.0
	Parous	117	90.0
Menopausal status	Pre-menopausal	68	52.3
	Post-menopausal	62	47.7
History of tubal sterilization	Yes	38	29.2
	No	92	70.8
History of oral contraceptive pill use	Yes	10	7.7
	No	120	92.3
History of breastfeeding	Yes	117	90.0
	No	13	10.0

Further inquiry into gynecological history revealed two (1.8%) patients with endometriosis and one with a history of infertility. No patients were found to be on hormone replacement therapy or ovulation-inducing drugs in the past. A family history of malignancy was documented in 11 patients (8.5%) (Table 3).

**Table 3:** Family history of cancer among the study participants (n=130)

Type of cancer	No. of patients	Percentage (%)
Breast cancer	5	3.8
Ovarian cancer	3	2.3
Other cancers	3	2.3
Total	11	8.5

Advanced disease was common, with FIGO stage III in 51 patients (39.2%) and stage IV in 28 patients (21.5%) (Table 4).

**Table 4:** FIGO staging of ovarian cancer among the study participants (n=130) FIGO, International Federation of Gynecology and Obstetrics

FIGO stage	No. of patients	Percentage
I	46	35.4
II	5	3.8
III	51	39.2
IV	28	21.5

Further analysis revealed no significant association between socioeconomic status and FIGO staging (p=0.53). Similarly, no statistically significant association was observed between FIGO stage and menopausal status (p=0.27) or parity status (p=0.18) (Tables 5-7).

**Table 5:** Association between FIGO stage and socioeconomic status *Chi-square test FIGO, International Federation of Gynecology and Obstetrics

FIGO stage	Upper, n (%)	Upper-middle, n (%)	Lower-middle, n (%)	Upper-lower, n (%)	Lower, n (%)	Total (n)	P-value
Stage I	3 (6.5)	11 (23.9)	20 (43.5)	7 (15.2)	5 (10.9)	46	0.53*
Stage II	0 (0)	0 (0)	5 (100)	0 (0)	0 (0)	5	
Stage III	0 (0)	12 (23.5)	27 (52.9)	5 (9.8)	7 (13.7)	51	
Stage IV	1 (3.6)	7 (25.0)	13 (46.4)	2 (7.1)	5 (17.9)	28	

**Table 6:** Association between FIGO stage and menopausal status *Chi-square test FIGO, International Federation of Gynecology and Obstetrics

FIGO stage	Premenopausal, n (%)	Postmenopausal, n (%)	Total (n)	P-value
Stage I	18 (39.1)	28 (60.9)	46	0.27*
Stage II	4 (80.0)	1 (20.0)	5	
Stage III	25 (49.0)	26 (51.0)	51	
Stage IV	15 (53.6)	13 (46.4)	28	

**Table 7:** Association between FIGO stage and parity *Chi-square test FIGO, International Federation of Gynecology and Obstetrics

FIGO stage	Nulliparous, n (%)	Parous, n (%)	Total (n)	P-value
Stage I	8 (17.4)	38 (82.6)	46	0.18
Stage II	0 (0)	5 (100)	5	
Stage III	4 (7.8)	47 (92.2)	51	
Stage IV	1 (3.6)	27 (96.4)	28	

Abdominal pain and distension were reported by approximately 94% of patients. Median (IQR) CA-125 level was 552.5 (171.8-1538) U/mL, with elevated levels (>35 U/mL) in 95% of patients. Median (IQR) RMI was 1881 (651-5477.3), and 121 patients (93.0%) had RMI>200. Epithelial tumors accounted for 114 cases (87.7%), of which serous adenocarcinoma was the most common subtype (76 cases, 58.5%). High-grade tumors were identified in approximately 63% of patients. Table 8 shows the various histologic types and subtypes in detail.

**Table 8:** Histopathological classification of ovarian cancer types among study participants (n=130)

Histological type	Subtype	No. of patients	Percentage (%)
Epithelial tumors	Serous	76	58.5
	Mucinous	29	22.3
	Endometrioid	4	3.1
	Clear cell	3	2.3
	Undifferentiated	2	1.5
Germ cell tumors	Teratoma	4	3.1
	Dysgerminoma	3	2.3
	Yolk sac tumor	4	3.1
Sex-cord stromal tumors	Adult granulosa cell tumor	5	3.8

Cytoreductive surgery alone was performed in 25 patients (19.2%), surgery followed by adjuvant chemotherapy in 11 patients (8.5%), neoadjuvant chemotherapy (NACT) followed by interval debulking in 48 patients (36.9%), and palliative chemotherapy in 33 patients (25.4%). Fertility-sparing unilateral salpingo-oophorectomy was performed in 13 (10.0%) patients, with a breakdown of 10 cases (7.7%) as standalone procedures, two cases (1.5%) involving adjuvant chemotherapy, and one case (0.8%) with NACT. Table 9 summarizes the treatment modalities used in the study.

**Table 9:** Treatment modalities and management strategies employed among study participants with ovarian cancer (n=130) ACT, adjuvant chemotherapy; NACT, neoadjuvant chemotherapy

Treatment modality	No. of patients	Percentage (%)
Cytoreductive surgery	25	19.2
Cytoreductive surgery followed by ACT	11	8.5
NACT followed by cytoreductive surgery	48	36.9
NACT followed by fertility-sparing surgery	1	0.8
Fertility-sparing surgery	10	7.7
Fertility-sparing surgery followed by ACT	2	1.5
Palliative chemotherapy	33	25.4

## Discussion

This study investigated the clinical, demographic, and histopathological characteristics of 130 histopathologically confirmed OC patients over a six-year period, along with their management strategies. Our findings offer valuable insights into the presentation patterns and treatment landscape of OC within our institutional setting.

Demographic and socioeconomic characteristics

The median age at presentation in our cohort was 50.5 years, with half of the patients falling within the 40- to 59-year age bracket. This age distribution is largely consistent with national and international epidemiological data, which typically report OC as a disease predominantly affecting perimenopausal and post-menopausal women. Recent global data suggest that the maximum burden of OC occurs in the age group of 55-59 years [14]. However, there is significant regional disparity among different socio-demographic index (SDI) regions, with high SDI regions showing a higher median age distribution extending into the late sixties [14], whereas Indian epidemiological data reveal a lower median age distribution of around 50 years [3,15-17]. This disparity underscores the need to screen age groups from 45 to 55 years in our region to reduce disease impact and improve outcomes.

Another notable finding of this study is that half of the patients belonged to the lower-middle socioeconomic class, highlighting the influence of social determinants of health on OC presentation in this region. Socioeconomic disadvantage is associated with limited health literacy, reduced awareness of early symptoms, financial constraints, and restricted access to timely diagnostic and specialist care. These factors contribute to delays in diagnosis and referral, increasing the likelihood of advanced-stage disease at presentation. Additionally, inequities in access to comprehensive oncologic services may further adversely affect outcomes, underscoring the need for targeted public health strategies to address structural barriers and improve equity in OC care.

Reproductive history and risk factor profile

Analysis of obstetric and gynecological history revealed that 10% of patients were nulliparous and 47.7% were postmenopausal, both recognized risk factors for OC [9]. Surprisingly, 90% of patients in our study reported breastfeeding their children, a practice generally associated with a reduced risk of OC. A recent meta-analysis indicates a significant protective effect for breastfeeding that increased with longer total breastfeeding duration [18]. The current study did not compare the breastfeeding data with the general population to perform a risk analysis.

The use of OCPs, also a known protective factor, was notably low at only 7.7%. A recent study demonstrated a significant impact of OCP use on the reduction of OC incidence in White women, followed by Black African and Asian women [19]. This low OCP utilization rate, coupled with potentially higher parity in the remaining cohort, highlights a complex interplay of risk and protective factors. Additionally, 29.2% of patients had undergone tubal ligation, a procedure increasingly recognized for its protective effect against some epithelial OCs, particularly serous types, by potentially preventing carcinogenic agents from reaching the ovaries or removing fimbrial precursor lesions [20]. However, our study does not have a control group to demonstrate the effect of tubal ligation on ovarian malignancy.

While a history of endometriosis is recognized to increase the risk of OC by 1.5-2 times, particularly for endometrioid and clear-cell subtypes [21], our study observed two patients with endometriosis who presented with serous type OC. This finding warrants further consideration, as it diverges from the commonly reported associations with nonserous histology.

A family history of malignancy was present in 8.5% of cases, which, while not a majority, underscores the importance of a comprehensive family history assessment for genetic predisposition, aligning with the prevalence of hereditary OC syndromes in a subset of patients globally. According to the literature, these syndromes, predominantly linked to BRCA1/2 mutations (hereditary breast and ovarian cancer or HBOC), constitute 10-15% of all OCs. BRCA1 carriers face a 40-50% risk, and BRCA2 carriers a 20-30% risk. Lynch syndrome, involving MMR genes such as MLH1/MSH2, accounts for 10-15% of hereditary cases with an approximate 8% lifetime risk. While other genes like PALB2, CHEK2, ATM, and RAD51C also contribute, BRCA mutations remain the most frequent genetic cause [22-24]. However, genetic analysis of participants was not performed in this study.

Clinical presentation and diagnostic markers

A critical and concerning observation was the advanced stage of presentation, with 60.7% of patients diagnosed at FIGO stages III (39.2%) and IV (21.2%). This high rate of late-stage diagnosis is a persistent challenge in OC worldwide, attributable to the insidious nature of symptoms and the lack of effective population-wide screening tools [25]. Consistent with this, approximately 94% of our patients presented with nonspecific symptoms such as abdominal distention and pain, which are typically associated with advanced disease when significant tumor burden or ascites has developed. The median CA-125 level was markedly elevated at 552.5 U/mL, with 95% of patients showing levels above the clinical threshold of 35 U/mL. This reinforces CA-125's utility as a diagnostic marker in symptomatic patients and its role in monitoring disease progression, though its limitations as a screening tool for asymptomatic individuals are well-documented [26]. Furthermore, the high median RMI score of 1,881, coupled with 93% of patients having values above the cutoff value of 200, demonstrates its effectiveness in triaging patients and identifying those at high risk of malignancy, thereby guiding referral to specialized gynecologic oncology centers for optimal management [13].

Despite the predominance of advanced-stage disease at presentation, the present study did not demonstrate a statistically significant association between FIGO stage and socioeconomic status, menopausal status, or parity. This suggests that while these demographic and reproductive factors are important determinants of OC risk, stage at diagnosis in this population may be more strongly influenced by delayed symptom recognition, nonspecific clinical presentation, and healthcare system-level barriers to early referral, rather than individual demographic characteristics alone. These findings reinforce the need for system-level interventions aimed at earlier detection and streamlined referral pathways, particularly in resource-limited settings.

Histopathological spectrum

Our study confirmed the global predominance of epithelial OCs, accounting for 87.7% of all cases. Serous adenocarcinoma was the most common histopathological subtype, representing 58.5% of all tumors, a finding consistent with international literature [2]. The aggressive nature of these tumors was further evidenced by the fact that 63% were classified as high-grade. The remaining 8.5% of cases were germ cell tumors, which typically present in younger patients and often have a better prognosis than epithelial subtypes [27]. This histopathological distribution informs our understanding of the biological characteristics and aggressive potential of OCs presenting in our patient population, emphasizing the need for tailored therapeutic approaches.

Treatment modalities and management strategies

**Figure 1:**
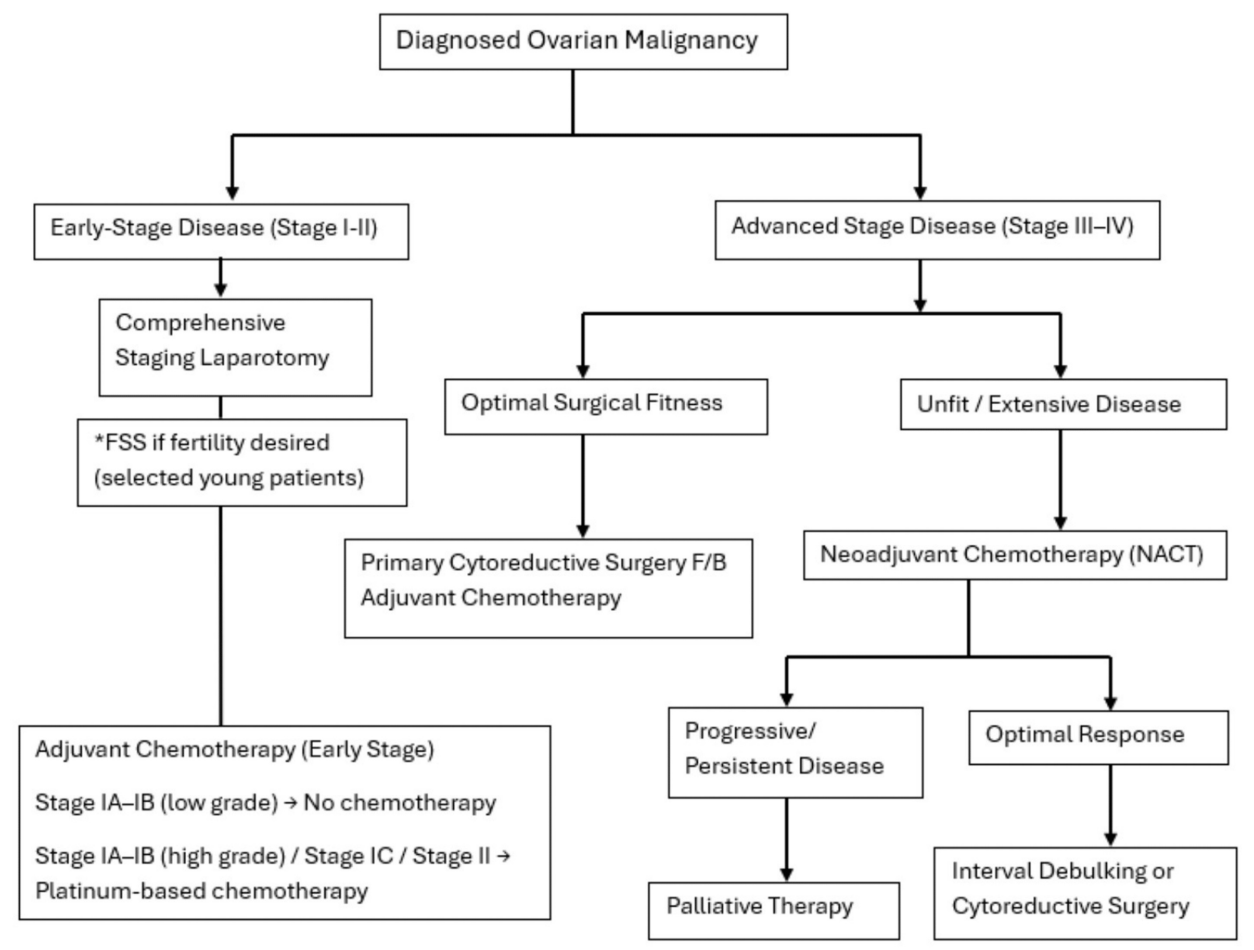
Institutional algorithm for the management of ovarian cancer *FSS, fertility-sparing surgery

Cytoreductive surgery, encompassing hysterectomy, salpingo-oophorectomy, and omentectomy with comprehensive staging, remained the cornerstone of management, reflecting established guidelines [10]. The diverse treatment landscape included surgery alone in 19.2% of patients (early-stage cases), followed by adjuvant chemotherapy in 8.5%. A substantial proportion (36.9%) underwent NACT followed by interval debulking surgery. This high rate of NACT is often indicative of advanced disease at presentation, where primary debulking is not feasible or safe, aligning with our finding of a high proportion of late-stage cases. Unfortunately, 25.4% of patients received only palliative chemotherapy, underscoring the severity and advanced nature of the disease in a significant subset of the cohort. Additionally, fertility-sparing surgery (FSS) with or without chemotherapy was performed in 10% of patients, primarily in younger individuals with early-stage disease and specific histologies. FSS offers fertility preservation by conserving the uterus and part of an ovary, and the staging involves removing the affected ovary, omentectomy, biopsies, and potentially lymph node assessment [28]. Beyond reproductive benefits, it avoids negative sequelae associated with surgical menopause. According to National Comprehensive Cancer Network (NCCN) guidelines, FSS is recommended for select unilateral stage I (IA, IC, not IB) and/or low-risk ovarian tumors (early-stage, grade 1, borderline) if fertility is desired and technically feasible [29]. These treatment patterns illustrate a contemporary approach to OC management, adapting to the complexities of disease presentation and patient factors. Figure 1 outlines our institutional workflow for OC management.

Strengths and limitations

The strengths of this study lie in its comprehensive six-year data collection, which provides a robust and detailed overview of the demographic profile, disease characteristics, and treatment patterns of OC patients managed at our institution. The relatively long study period enhances the reliability of observed trends and reflects real-world clinical practice in a tertiary-care setting.

Nevertheless, several limitations must be acknowledged. First, the single-center design, conducted at a tertiary-care referral hospital, introduces the potential for selection bias, as such centers often manage more advanced or complex cases; consequently, the findings may not be fully generalizable to the broader population. Multicenter studies would be valuable to validate and extend these observations. Second, although the study provides detailed information on clinicopathological variables and management strategies, survival outcomes, such as overall survival and progression-free survival, were not assessed, limiting conclusions regarding long-term treatment effectiveness. Third, the absence of genetic and molecular profiling data, including for BRCA mutations and other hereditary cancer syndromes, restricts the ability to correlate molecular characteristics with treatment decisions and outcomes, which is increasingly important in contemporary OC management. Despite these limitations, the study offers meaningful insights into institutional patterns of OC presentation and management. Addressing these gaps in future research would further strengthen the evidence base and enhance the applicability of findings.

## Conclusions

In conclusion, this hospital-based study provides important insights into the epidemiological profile, clinical presentation, histopathological spectrum, and management patterns of OC in eastern India, a region with limited published data. The findings reaffirm that OC in this setting predominantly affects women in the perimenopausal age group and is characterized by a high burden of advanced-stage disease at diagnosis, likely driven by socioeconomic disparities, limited awareness, and the nonspecific nature of early symptoms. The predominance of high-grade epithelial tumors and the substantial proportion of patients requiring neoadjuvant or palliative chemotherapy further underscore the aggressive disease biology and delayed presentation. Furthermore, the consistent utility of ultrasonography, CA-125, and the RMI in identifying high-risk cases supports their continued use as pragmatic diagnostic and triage tools in resource-constrained settings. Adherence to standard surgical and chemotherapeutic protocols, including the judicious use of NACT and FSS in selected patients, reflects contemporary evidence-based practice within our institution.

Looking ahead, future research should prioritize prospective, multi-institutional studies across eastern India to validate these findings, improve regional representativeness, and enable meaningful comparisons of outcomes. There is a critical need to incorporate survival analysis, quality-of-life measures, and treatment-related morbidity into future studies to better evaluate the long-term effectiveness of different management strategies. Additionally, integration of genetic testing and molecular profiling, particularly for BRCA mutations and other hereditary cancer syndromes, could help refine risk stratification, guide targeted therapies, and inform family counseling. From a public health perspective, targeted awareness programs, capacity building of primary care providers for early symptom recognition, and strengthened referral pathways to specialized gynecologic oncology centers are essential to shift diagnosis toward earlier stages. Policymakers should also consider region-specific strategies to address socioeconomic barriers to timely care. Collectively, such efforts may contribute to earlier detection, personalized treatment approaches, and ultimately improved outcomes for women with OC in this underserved region.
